# Effects of short-chain fatty acids in inhibiting HDAC and activating p38 MAPK are critical for promoting B10 cell generation and function

**DOI:** 10.1038/s41419-021-03880-9

**Published:** 2021-06-07

**Authors:** Fagui Zou, Yi Qiu, Yilian Huang, Hang Zou, Xiao Cheng, Qingru Niu, Aoxiang Luo, Jianbo Sun

**Affiliations:** 1 Guanghua School of Stomatology, Hospital of Stomatology, Sun Yat-Sen University, Guangzhou, 510055 China; 2 Guangdong Provincial Key Laboratory of Stomatology, Guangzhou, 510055 China; 3 Zhongshan School of Medicine, Sun Yat-Sen University, Guangzhou, 510080 China; 4 School of Nursing, Guangdong Pharmaceutical University, Guangzhou, 510006 China

**Keywords:** Cell signalling, B cells, Inflammatory diseases

## Abstract

B10 cells are regulatory B cells capable of producing IL-10 for maintaining immune homeostasis. Dysregulation of B10 cells occurs in autoimmune and inflammatory diseases. Modulation or adoptive transfer of B10 cells is a promising therapeutic strategy. The short-chain fatty acids (SCFAs), the metabolites of microbiota, play a critical role in maintaining immune homeostasis and are the potential drugs for the modulation of B10 cells. It is not clear whether and how SCFAs upregulate the frequency of B10 cells. Here, we found that SCFAs could promote murine and human B10 cell generation in vitro. Upregulation of B10 cells by butyrate or pentanoate was also observed in either healthy mice, mice with dextran sodium sulfate (DSS)-induced colitis, or mice with collagen-induced arthritis. Moreover, SCFA treatment could ameliorate clinical scores of colitis and arthritis. Adoptive transfer of B cells pretreated with butyrate showed more alleviation of DSS-induced colitis than those without butyrate. A further study demonstrates that SCFAs upregulate B10 cells in a manner dependent on their histone deacetylase (HDAC) inhibitory activity and independent of the G-protein-coupled receptor pathway. Transcriptomic analysis indicated that the MAPK signaling pathway was enriched in B10 cells treated with butyrate. A study with inhibitors of ERK, JNK, and p38 MAPK demonstrated that activating p38 MAPK by butyrate is critical for the upregulation of B10 cells. Moreover, HDAC inhibitor has similar effects on B10 cells. Our study sheds light on the mechanism underlying B10 cell differentiation and function and provides a potential therapeutic strategy with SCFAs and HDAC inhibitors for inflammation and autoimmune diseases.

## Introduction

Regulatory B cells (Bregs) are immunosuppressive B lymphocytes capable of secreting immunomodulatory cytokines such as interleukin-10 (IL-10), IL-35, and transforming growth factor-beta (TGF-β)^1–4^. Breg deficiency or dysfunction has been reported in many autoimmune or inflammatory disorders in both humans and mice^5^. B10 cells are the most studied and major subpopulation of Bregs, which are capable of producing IL-10 for immune suppression^6^. B10 cells contribute to the maintenance of the immune homeostasis and immunological tolerance, mostly by inhibiting the expansion of pro-inflammatory lymphocytes such as T-helper type 1 (Th1) and Th17 cells or favoring regulatory T cell (Treg) differentiation via IL-10 and TGF-β signals. Previous studies have suggested that B cell activation signals such as B cell receptor engagement, CD40 ligation, Toll-like receptor pathway, or inflammatory signals played crucial roles in Breg expansion or differentiation^7,8^. The molecular mechanism underlying the regulation and differentiation of B10 cells is still unclear, although IL-10 production by other immune cells has been linked to the MAPK and PI3K/AKT signaling pathway^9^.

It has been reported that modulation or adoptive transfer of B10 cells could ameliorate periodontitis and collagen-induced arthritis (CIA)^10^. Enhancement of B10 cell generation in vivo with drugs is a promising strategy in the therapy of autoimmune and/or inflammatory diseases. Short-chain fatty acids (SCFAs), the end metabolites of microbiota through anaerobic fermentation, may be such candidates for inducing B10 cells. SCFAs have no more than six carbons and contain acetate (C2), propionate (C3), butyrate (C4), pentanoate (C5), and hexanoic (C6), of which the first three represent 90–95% of the total SCFAs present in the colons^11,12^. SCFA functions as an essential mediator between the commensal microbiota and immune system and regulates the immune response by acting as histone deacetylase inhibitor (HDACi) or signaling through G-protein-coupled receptor (GPCR) pathway mainly^13^. In addition, SCFAs could promote Treg cell differentiation and suppress the pathogenic Th1/17 cell differentiation in many autoimmune disease models^14,15^. Moreover, the microbiota has been proven to govern the differentiation of Bregs, and perturbation of the gut microbiome impaired the frequency and function of Bregs^16^. Several studies demonstrate that SCFAs could enhance B10 cell generation through different mechanisms^17–19^. But another recent study reported that SCFA butyrate does not alter the frequency or number of splenic Breg subsets in vivo, although it could promote B10 cell function^20^. It is unclear whether SCFAs could alter B10 cell generation in other tissues or peripheral blood^20^, and the mechanism that SCFAs promote B10 cell generation needs further study. Hence, we studied here whether and how SCFAs upregulate the differentiation and function of B10 cells.

## Results

### SCFAs promote B10 cell generation in vitro

**Fig. 1:**
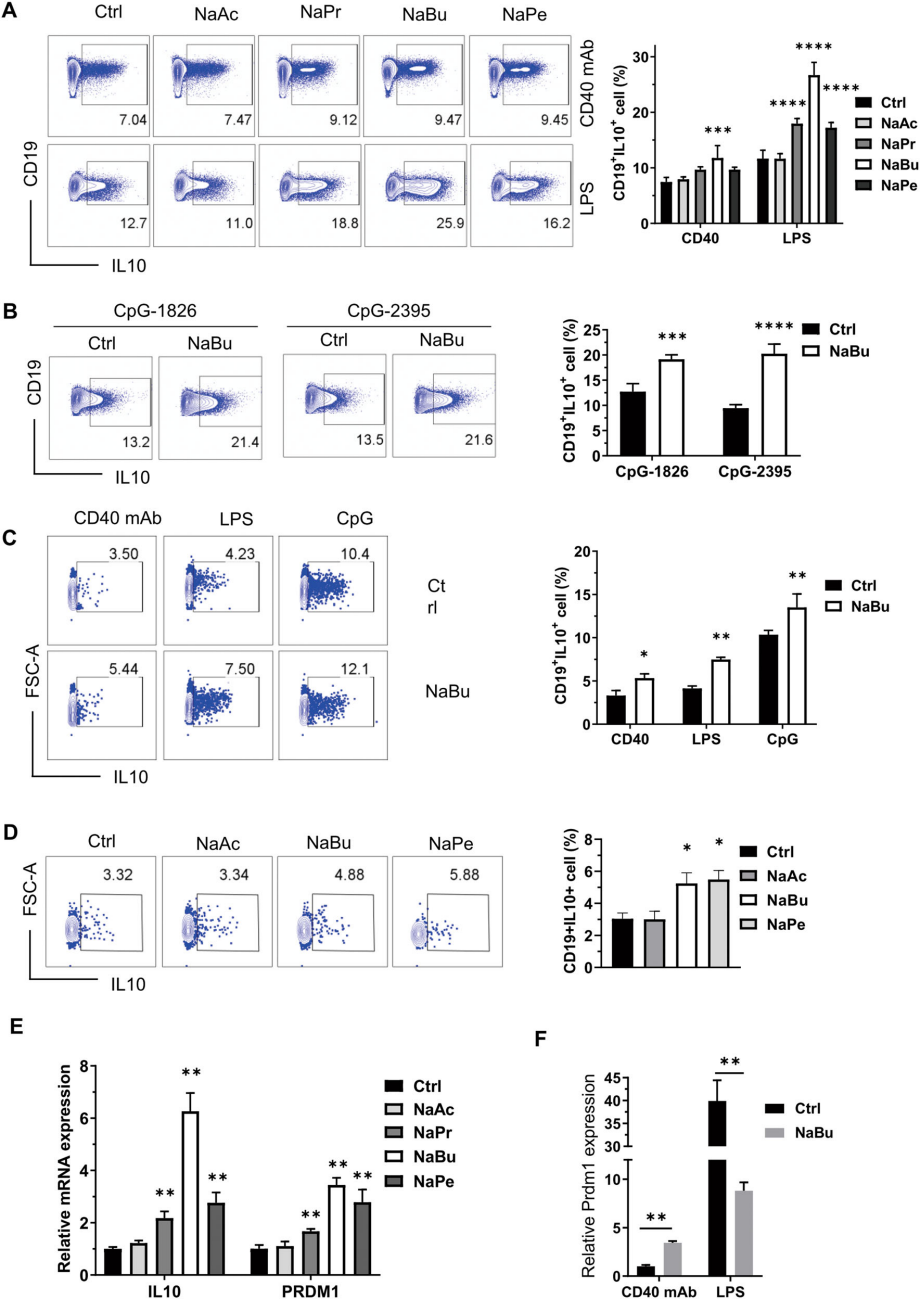
SCFAs promote both murine and human B10 cell generation in vitro. **A** Representative FACS plots of splenic B cells purified from C57BL/6J mice and cultured with murine CD40 mAb or LPS in the absence or presence of corresponding SCFAs (0.5 mM) for 48 h. The right bar graph is a statistical result of B10 cell percentage. **B** Representative FACS plots of murine splenic B cells cultured with CpG-1826 or CpG-2395 and incubated with or without NaBu. **C** Representative FACS plots of human PBMCs stimulated by LPS, CpG-ODN 2395, or human CD40 mAb with or without sodium butyrate (0.5 mM) for 48 h. **D** Representative FACS plots of human PBMCs stimulated by human CD40 mAb with or without 0.5 mM of NaAc, NaBu, or NaPe for 48 h. **E** Statistical mRNA level of IL-10 and PRDM1 detected by RT-qPCR in purified murine B cells cultured with or without SCFAs under the existence of CD40 mAb for 48 h. **F** Statistical mRNA level of PRDM1 detected by RT-qPCR in purified murine B cells cultured with or without SCFAs under the existence of CD40 mAb or LPS for 48 h. The data are presented as mean ± SD from at least three independent experiments. *P < 0.05, **p < 0.01, ***p < 0.001, and ****p < 0.0001 compared to Ctrl with the same stimuli.

SCFAs could suppress inflammation by promoting Breg function or differentiation, but the results of B10 cell induction by SCFAs remain controversial as mentioned above. Thus, we checked whether SCFAs could enhance B10 cell generation and function. First, we checked the effects of CD40 monoclonal antibody (mAb), CpG, and lipopolysaccharide (LPS), the stimuli used usually for B cell culture, on B10 cell differentiation via BCR, TLR9, and TLR4 signaling pathways, respectively. The effects varied when B cells were treated with different stimuli (Figure S1A). The mean fluorescence intensity of IL-10 is much higher, although the proportion of IL-10^+^ cells is lower in B cells treated with CD40 mAb than LPS or CpG (Figure S1B). Under the existence of CD40 mAb, which is used mostly for analysis of B10 progenitor (B10pro) plus B10 cells, incubation in culture for 48 h with 500 μM propionate, butyrate, and pentanoate, but not acetate, could significantly enhance the percentage of B10 cells (B10%) (Fig. 1A). SCFAs, especially butyrate, showed very high induction efficacy of B10 cells when cells were stimulated by LPS or CpG (Fig. 1A, B and Figure S1D). Unlike the reported result that butyrate causes obvious cell apoptosis at a concentration of 5 mM, butyrate at 0.5 mM did not cause cell death significantly, although it inhibited B cell proliferation (Figure S1C, D) and showed the highest efficiency on B10 cell induction. Similarly, human B10 cell generation could be enhanced by sodium butyrate (NaBu) when human peripheral blood mononuclear cells (PBMCs) were cultured with CD40 mAb, LPS, or CpG (Fig. 1C). Treatment with NaBu, but not acetate, enhanced human B10 cell generation as well as pentanoate when the cells were stimulated with CD40 mAb (Fig. 1D).

It has been reported that Prdm1 promotes IL-10 expression in B cells^21^ when stimulated with CpG, while Prdm1-deficient mice have more IL-10^+^ B cells^22^. Butyrate hinders B cell differentiation into plasmablasts^20^, in which Prdm1 plays a vital role^23^. In our experiment, Prdm1 as well as IL-10 showed increased messenger RNA (mRNA) levels in the presence of SCFAs, except acetate, when cultured with CD40 mAb (Fig. 1E). It is interesting to note that butyrate reduces Prdm1 expression in B cells stimulated with LPS, while it enhances the Prdm1 expression in CD40 mAb-stimulated B cells, which show higher IL-10 expression on a per-cell basis (Figure S1B), but much lower Prdm1 expression than those stimulated by LPS (Fig. 1F). These results suggest that Prdm1 and butyrate play complex roles in IL-10 production and B10 cell differentiation.

**Fig. 2:**
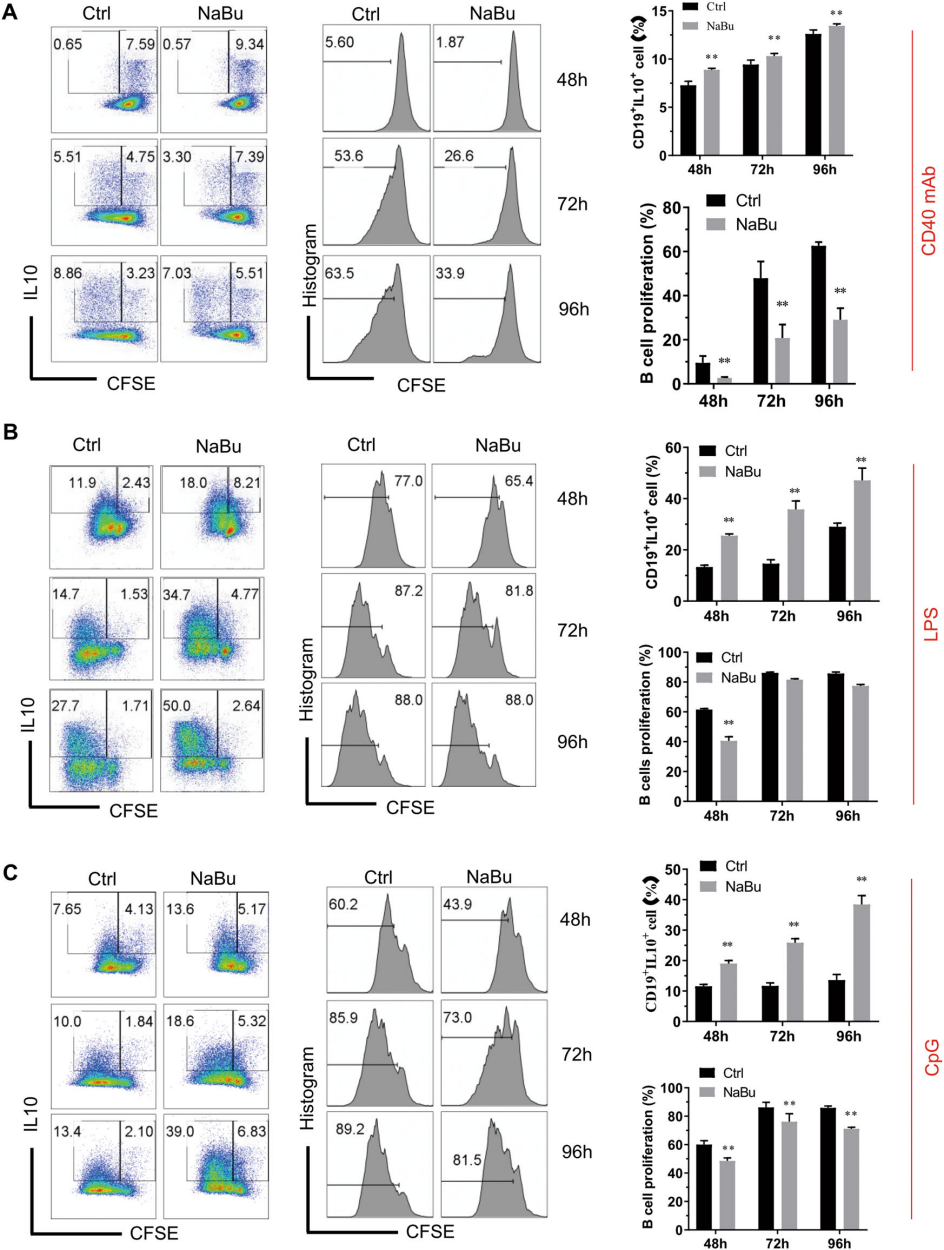
Sodium butyrate inhibits cell proliferation and promotes B10 cell differentiation. Murine splenic B cells were isolated and labeled with CFSE and then cultured with CD40 mAb (**A**), LPS (**B**), or CpG-ODN 2395 (**C**) in the presence or absence of sodium butyrate for 48 h. Representative FACS plots of IL-10 secretion and CFSE dilution, followed by proliferation of B cells were shown in the left and middle panels, while the right panels showed the statistical results. The data are presented as mean ± SD from three independent experiments. **P < 0.01 compared to Ctrl at the same culture time.

To find out whether the enhancement of B10% by SCFAs resulted from promoting differentiation of B10^+^ cells, we checked the proliferation rate of B cells treated with or without NaBu plus CD40 antibody, LPS, or CpG for 48, 72, and 96 h. NaBu treatment showed inhibition of cell proliferation in culture conditions with CD40 mAb or CpG for all the culture time (Fig. 2A, B). There are significant differences in B10% between control and NaBu treatment for all the culture time in the culture with CD40 mAb or CpG. The B10% increased following the increase of the culture time. Under stimulation with LPS, B cells showed a similar phenotype as those stimulated with CD40 mAb and CpG after treatment with NaBu for 48 h, but showed no cell proliferation inhibition, while it showed much higher B10% compared with the control after treatment with NaBu for 72 and 96 h (Fig. 2C). Moreover, most B10^+^ cells appeared in the population proliferating well in both control and NaBu treatment, which is not surprising because cells growing better have higher cell metabolism and activities, including cytokine secretion most of the time. Taken together, these data suggest that NaBu promotes B10 cell differentiation since the cells with inhibited proliferation showed much fewer B10 cells than those proliferating well.

### SCFAs promote B10 cell generation in vivo

**Fig. 3:**
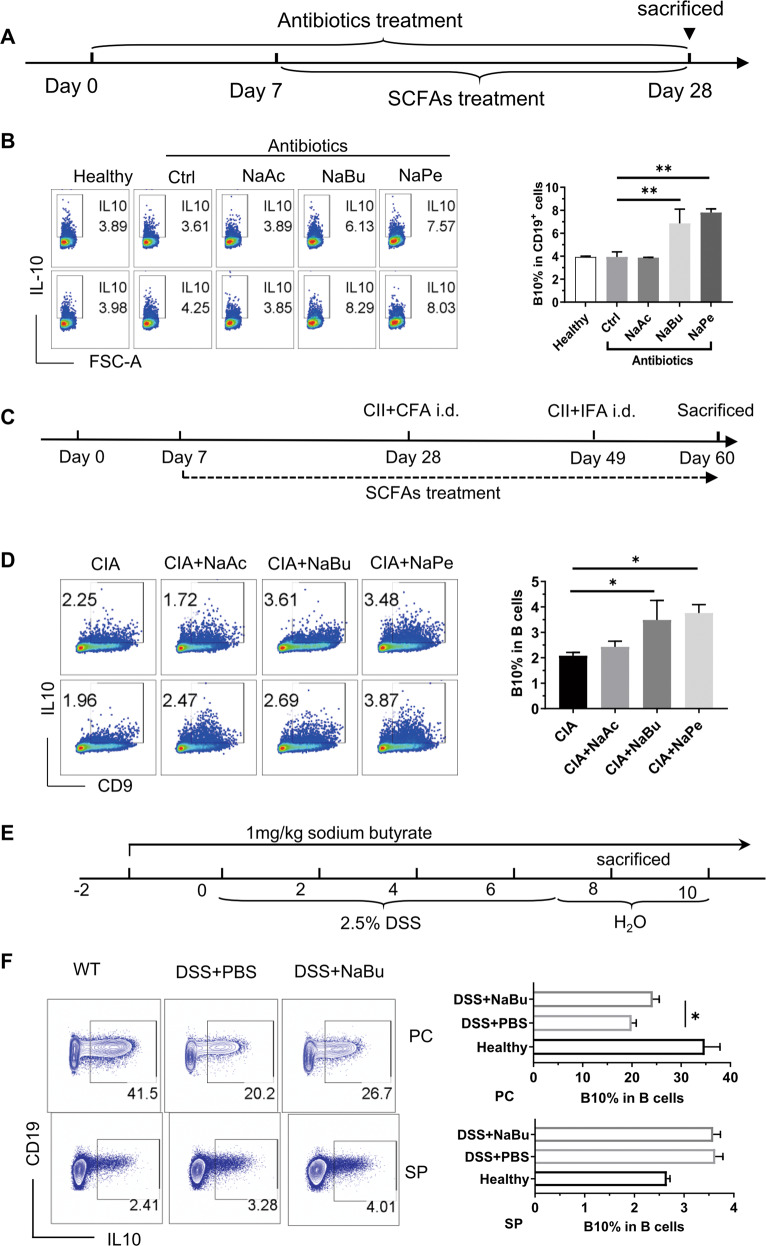
SCFAs promote B10 cell generation in vivo. **A** Schematic diagram of the procedure adopted for treating C57BL/6J mice with SCFAs. Mice were randomly grouped and administered with antibiotics in drinking water for 7 days to clear the gut bacteria and then received SCFAs in drinking water containing antibiotics for 3 weeks before being sacrificed for sampling. **B** Representative FACS plots of IL-10^+^ cells in CD19^+^ B cells from PBMCs of mice treated as described in “Materials and methods” with the procedure of (**A**). Cells were cultured with L + PIM for 5 h before staining. The right bar graph is a statistical result of B10 cell percentage (similarly hereinafter). **C** Schematic diagram of procedure adopted for treating DBA/1J mice with SCFAs for prevention of collagen-induced arthritis. Mice were treated the same as in (**A**) before they received their first immunization with CII plus CFA on Day 28, then got the immune boost with CII plus IFA on Day 49, and finally they were sacrificed on Day 60. SCFAs were provided in the drinking water from Day 7 to 60. **D** Representative FACS plots of IL-10^+^ cells in CD19^+^ B cells from splenocytes of mice in (**C**). Cells were cultured with CD40 mAb for 48 h and L + PIM for the last 5 h. **E** Schematic diagram of procedure treating C57BL/6 mice with SCFAs for preventing colitis induced with DSS. **F** Representative FACS plots of IL-10^+^ cells in CD19^+^ B cells from the spleen or peritoneal cavity fluids of mice in (**D**). Cells were cultured with L + PIM for 5 h before staining. The data are presented as mean ± SD from at least six mice in each group. *P < 0.05 and **p < 0.01, compared to Ctrl (**B**), CIA (**D**) or DSS+PBS (**F**) as indicated.

Next, we checked whether SCFAs could enhance B10 cell generation in vivo. To eliminate the effects of SCFAs produced by gut microbiota, antibiotics were administered in drinking water before treatment with SCFAs and were continued until the end of the experiment (Fig. 3A). As shown in Fig. 3B, treatment with NaBu or NaPe significantly enhanced B10 cell frequency in B cells from PBMCs of healthy C57BL/6 mice treated with SCFAs in drinking water for 3 weeks. Similarly, in DBA1/J mice with CIA, supplementation with NaBu or NaPe enhanced significantly B10 cell frequency in splenic B cells, which were further cultured with CD40 mAb for 48 h for B10 cell maturation and stimulated with LPS, phorbol myristate acetate, ionomycin, and monensin (L + PIM) for the final 5 h (Fig. 3C, D), although it is consistent with the previous report^20^ that SCFAs showed no alteration on the B10 cell frequency in splenic B cells stimulated with L + PIM for 5 h only (Figure S2). Moreover, in C57BL/6 mice with dextran sodium sulfate (DSS)-induced colitis, NaBu treatment enhanced B10 cell frequency in B cells from the peritoneal cavity fluids but not in splenic B cells stimulated with L + PIM for 5 h only (Fig. 3E, F).

### SCFAs enhance B10 cell function

**Fig. 4:**
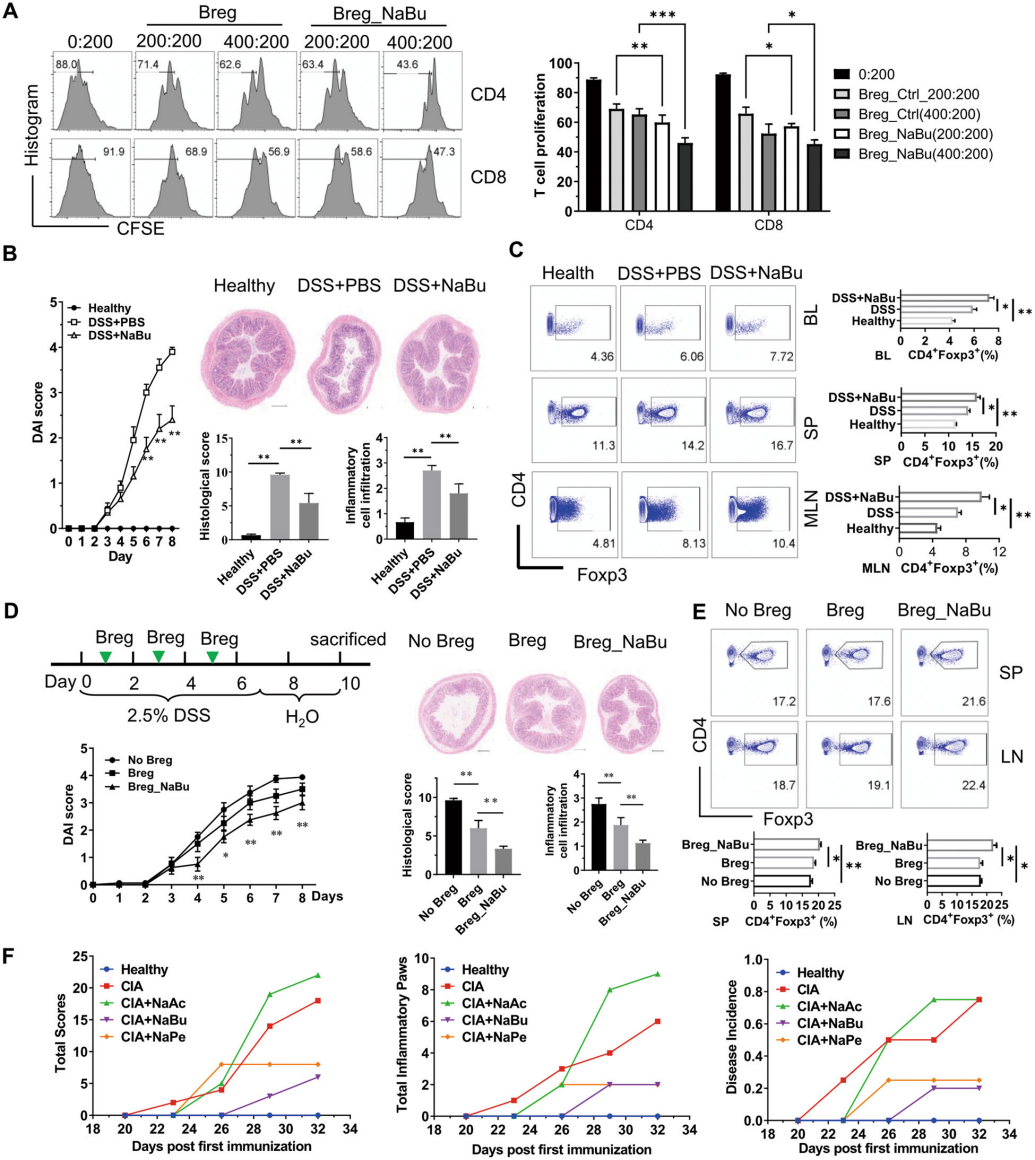
Sodium butyrate promotes B10 cell functions. **A** Inhibition of T cell proliferation by B cells. B cells isolated with CD19 microbeads were treated by LPS with or without sodium butyrate for 2 days and then washed twice with PBS before coculture for 72 h (100 or 200 × 10^3^) with fresh splenocytes (100 × 10^3^) at a ratio of 1:1 or 2:1 in a plate coated with CD3e and CD28 antibody. The left panel shows the representative FACS plots of T cells and the right panel shows the statistical results. **B** Inhibition of DSS-induced colitis by i.p. injection of NaBu daily with the same procedure as in Fig. 3E. The severity of colitis was evaluated by DAI scores and examination of histological colon sections (H&E stained). **C** Upregulation of Treg by NaBu in lymphocytes from peripheral blood, spleen, and LN of mice in (**B**). **D** Inhibition of DSS-induced colitis by i.p. injection (green arrows) of CD19^+^ B cells pretreated with NaBu for 48 h as shown in the schematic diagram. The severity of colitis was evaluated by DAI scores and examination of histological colon sections (H&E stained). **E** Upregulation of Treg by Breg transfer in lymphocytes from the spleen and LN of mice in (**D**). **F** Inhibition of CIA by NaBu treatment with the same procedure as in Fig. 3C. Scale bar represents 200 μm in each microscopy image. The data are presented as mean ± SD from at least six mice in each group or three independent experiments. *P < 0.05, **p < 0.01, and ***p < 0.001 compared to the healthy group (**B**), Breg (**D**), or as indicated.

To further evaluate the effect of SCFAs on the functional properties of B10 cells, we checked the inhibition effects of B cells with or without NaBu treatment on the proliferation of carboxyfluorescein succinimidyl ester (CFSE)-labeled T cells. The results showed that the suppressive function of Bregs on either CD4^+^ T cells or CD8^+^ T cells was enhanced when pretreated with butyrate (Fig. 4A). B10 cells play an important role in inflammation-related diseases. It has been found that DSS-induced colitis is more severe in B cell-deficient mice^24^, and could be mitigated by adoptive transfer of B10 cells from wild-type mice^25^. Then, we detected whether the butyrate-mediated upregulation of Bregs could suppress acute inflammation or chronic inflammation. As shown in the results, NaBu significantly reduced the severity of colitis (Fig. 4B); ameliorated the infiltration of inflammatory cells, loss of goblet cells, and the destruction of crypt architecture (Fig. 4B); and enhanced both B10% (Fig. 3F) and Treg cells (Fig. 4C). In addition, the expression of inflammatory cytokines, including IL-1β, IL-6, interferon-γ (IFN-γ), and tumor necrosis factor-α (TNF-α) in the colon, was decreased significantly after butyrate intervention (Figure S3A). To test whether the immunosuppressive function of NaBu resulted from upregulation of Bregs, we adoptively transferred Bregs polarized with or without NaBu in vitro for 2 days into DSS-induced colitis mice (Fig. 4D). The results showed that Bregs treated with butyrate enhanced the maintenance potential of B10 cells in the peritoneal cavity (Figure S3B) and ameliorated the severity of colitis (Fig. 4E). Treg cells from the spleen and mesenteric lymph nodes were significantly elevated, while the IL-6 and TNF-α in the colon were reduced in the butyrate treatment group (Fig. 4E and Figure S3C). Moreover, treatment with NaBu or NaPe significantly inhibited the development of arthritis induced by collagen (Fig. 4F). Taken together, these findings suggest that NaBu enhanced the immunoregulatory function of Bregs.

### Upregulation of B10 by SCFAs is independent of GPCR activity

**Fig. 5:**
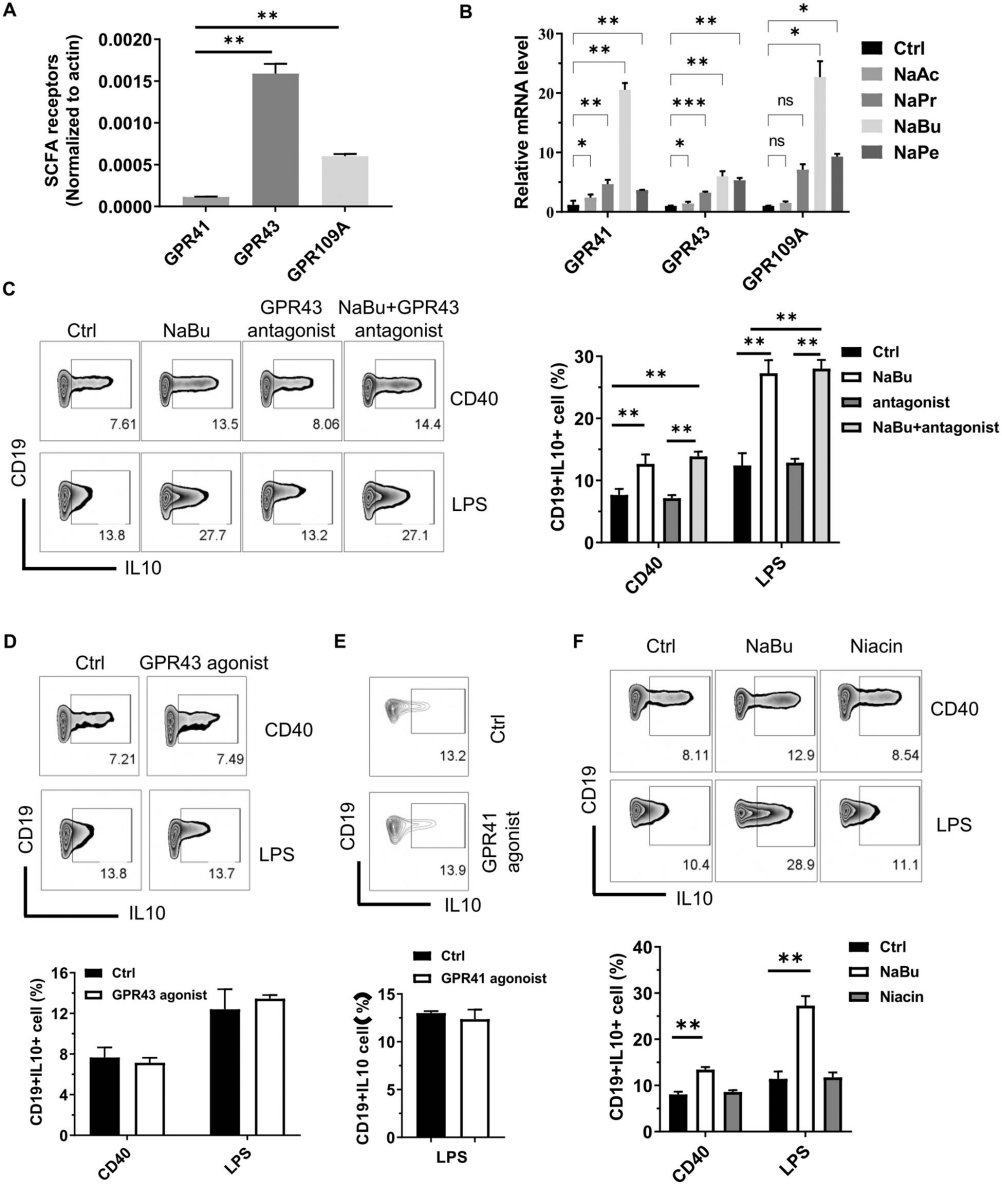
Promotion of B10 cell generation by SCFAs is independent of GPCR activity. **A**, **B** The mRNA expression of GPR41, GPR43, and GPR109A detected by RT-qPCR in murine splenic naive B cells (**A**) and B cells cultured with or without 500 μM SCFAs under the existence of CD40 mAb for 2 days (**B**). **C**–**F** B10 cell frequency in purified splenic B cells cultured with or without 500 μM NaBu, 10 μM GPR43 antagonist (**C**), 1 μM GPR43 agonist (**D**), 1 μM GPR41 antagonist AR420626 (**E**), or 1 mM GPR109A agonist niacin (**F**) under the existence of CD40 mAb or LPS for 2 days. The data are presented as mean ± SD from three independent experiments. *P < 0.05, **p < 0.01, and ***p < 0.001 compared between the groups as indicated.

It is important to find out how butyrate upregulates B10 cells. We first investigated whether butyrate upregulates B10 cells through its known functions of activating the GPCR pathway. The expression profiles of GPCRs detected by quantitative PCR (qPCR) indicate that naive B cells expressed a relatively high levels of GPR43 and GPR109A transcripts, but a very low level of GPR41 (Fig. 5A). Treatment with butyrate could significantly upregulate the expression of all detected GPCRs (Fig. 5B), which is consistent with the previous report^26^ that butyrate activates the downstream pathways through GPR41 or GPR109A as well as GPR43. However, blocking GPR43 signals by GLPG0974 (GPR43 antagonist) did not impair the upregulation of B10 by butyrate in vitro (Fig. 5C and Figure S4). Similarly, GPR43 agonist had no effects on B10 differentiation (Fig. 5D). Moreover, neither AR420626 (GPR41 agonist) nor niacin (GPR109A agonist) promoted B10 cell generation (Fig. 5E, F), although they demonstrated the activity enhancing calcium influx (Figure S4B). These results suggest that the upregulation of B10 cells by butyrate is independent of the activation of GPCR.

### HDAC inhibitory activity is critical for SCFAs to enhance B10 cell generation

**Fig. 6:**
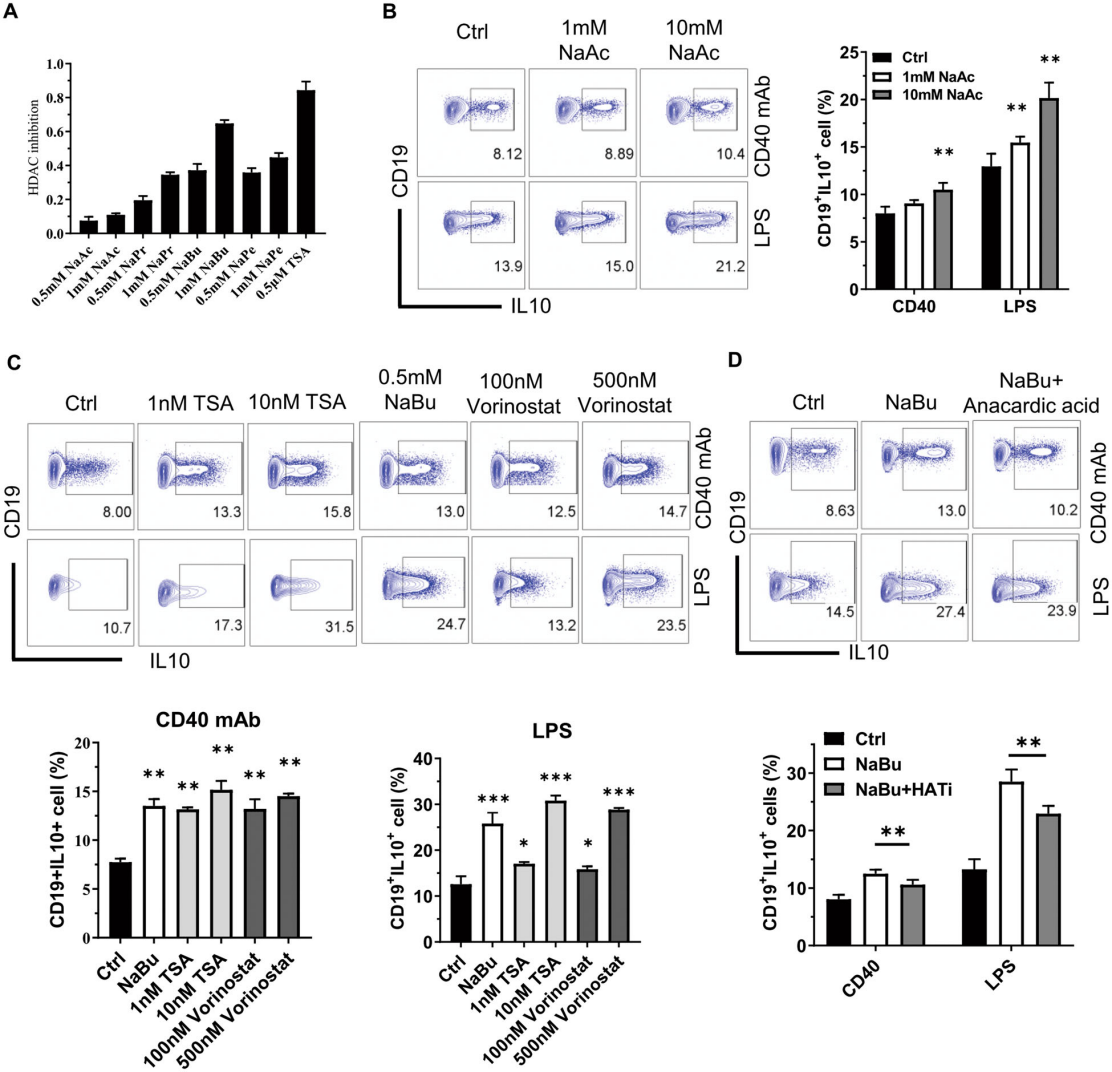
Promotion of B10 cell generation by butyrate depends on its HDAC inhibitory activity. **A** Purified splenic B cells were polarized to Bregs after a 2-day coculture with indicated SCFAs or Trichostatin A (TSA, an HDAC inhibitor with high efficiency) in the presence of LPS; the nuclear protein was prepared and tested for HDAC inhibition by ELISA. **B**–**D** B10 cell frequency in purified splenic B cells cultured with or without sodium acetate (**B**), HDAC inhibitors TSA or vorinostat (**C**), or HAT inhibitor (HATi) anacardic acid (**D**) under the existence of CD40 mAb or LPS for 2 days. Results were shown as representative FACS plots or bar graphs. The data are presented as mean ± SD from three independent experiments. *P < 0.05, **p < 0.01, and ***p < 0.001 compared to Ctrl or as indicated.

Afterward, we investigated whether butyrate promotes B10 cell generation through its inherent HDAC inhibitory activity^27^. In parallel with the results of B10 cell induction, the relative HDAC inhibitory activity ranked from butyrate, pentanoate, propionate to acetate in a dosage-dependent manner (Fig. 6A). Consistent with the fact that HDAC inhibitory potential is related to the concentration of SCFA^28^, acetate also significantly promoted B10 generation with the boosted concentration (Fig. 6B). Further study indicated that HDACis, either vorinostat or trichostatin A (TSA), promoted B10 cell generation in a dose-dependent manner (Fig. 6C). On the contrary, histone acetyltransferase (HAT) inhibitor can antagonize the B10 cell induction by butyrate (Fig. 6D). Taken together, these data demonstrate that HDAC inhibitory activity may be shared by SCFAs for the promotion of B10 cell generation.

### Activation of p38 MAPK is important for SCFAs/HDACi to promote B10 generation

**Fig. 7:**
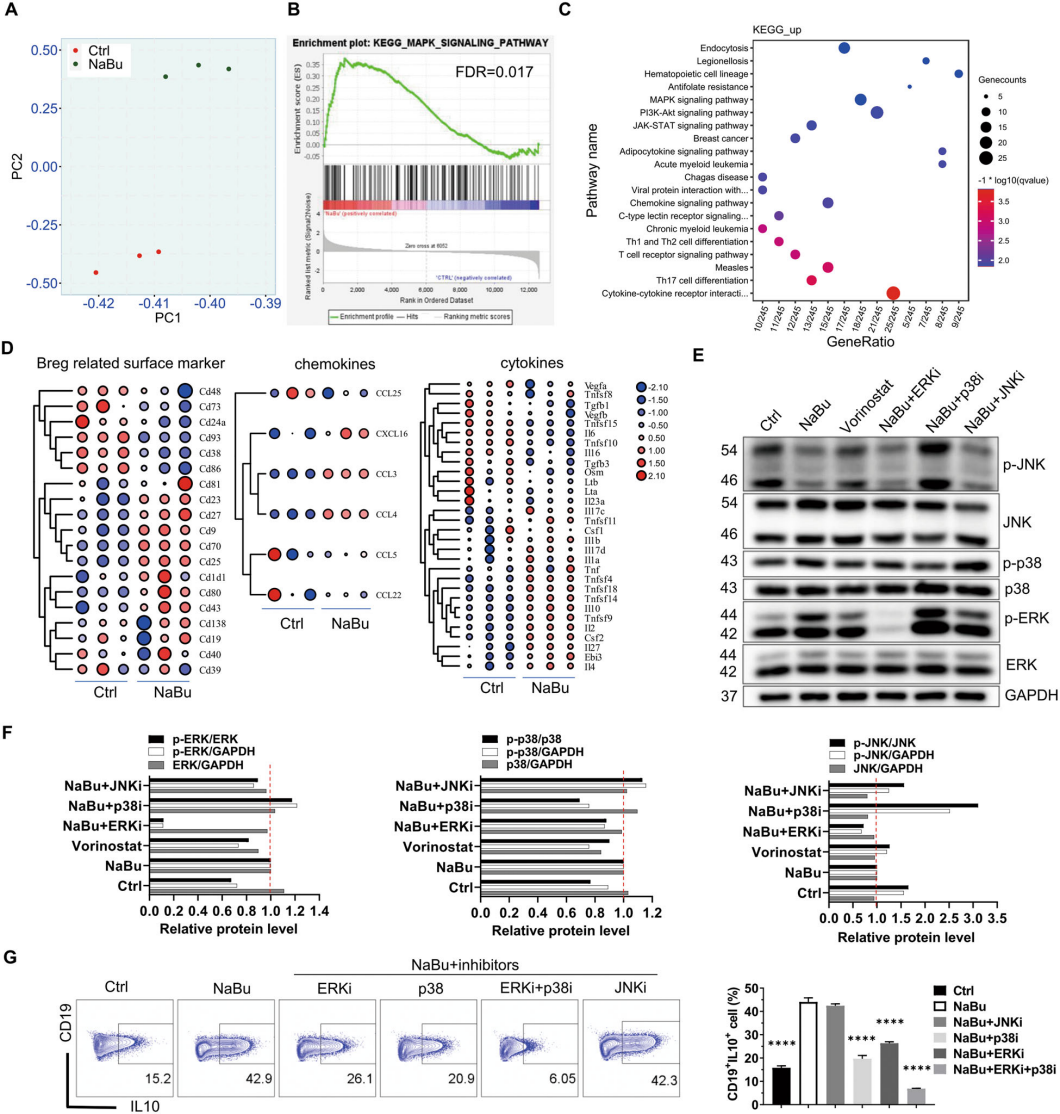
Promotion of B10 cell generation by butyrate and HDACi is dependent on ERK and p38 MAPK activity. Splenic B cells isolated from C57BL/6J mice were cultured with or without NaBu under the existence of LPS for 2 days and harvested for RNA-seq analysis. **A** The principal component analysis was performed on total normalized counts before statistical analysis. **B**, **C** Potential key pathways involved in the promotion of B10 cell generation by butyrate. DEGs identified as fold change >2 and *p* value of < 0.05 were used for gene set enrichment analysis (**B**) and KEGG pathway analysis (**C**). **D** Heatmap of the fold change of differentially expressed cytokines, chemokines, and Breg-related surface markers in cells treated with NaBu compared to the control. **E**–**G** Protein level evaluated by immunoblot analysis (**E**, **F**) and B10 cell frequency detected by flow cytometry (**G**) in B cells, which were cultured for 48 h in the presence of LPS with or without NaBu (1 mM) or MAPK inhibitors including ERKi (5 μM), p38i (5 μM), and JNIKi (1 μM). The relative protein levels in immunoblots were semi-quantified by the Image J software and the ratio between the level of phosphorylated and total target protein was calculated and visualized as bar graphs in (**F**). The data are presented as mean ± SD from three independent experiments. ****P < 0.0001 compared to NaBu.

To further understand the underlying mechanism by which SCFAs upregulate B10 cells, we performed transcriptomic analysis and signaling pathway intervention with inhibitors. Principal component analysis revealed that RNA profiling of the cells treated with NaBu is quite distinct from the control (Fig. 7A). There are 1462 differentially expressed genes (DEGs), of which 720 genes were upregulated and 742 genes downregulated, respectively (fold change > 2 and adjusted *p* value < 0.05) (Figure S5A and Table S1). Quantitative reverse transcription PCR (RT-qPCR) showed that butyrate enhanced IL-10 expression, while it suppressed IL-6 expression (Figure S5B, C), confirming the reliability of RNA-sequencing (RNA-seq) data. Furthermore, butyrate significantly changed the expression of cytokines, chemokine, and surface markers (Fig. 7D). Butyrate upregulated TNF-α, while it suppressed IL-6 secretion, which is consistent with the results of FACS (Figure S5D). Besides, some Breg-associated surface markers reported previously were upregulated by butyrate (Fig. 7D). Kyoto Encyclopedia of Genes and Genomes (KEGG) and Gene ontology enrichment indicated that the MAPK signaling pathway might be involved in the B10 cell upregulation by butyrate (Fig. 7C and Figure S5E), which was further supported by gene set enrichment analysis (GSEA) (Fig. 7B). To test whether butyrate and other HDACi upregulates B10 cells through the MAPK signaling pathway, B cells were treated with ERK1/2, p38, or JNK inhibitors in the presence of LPS and butyrate (Figure S6C). Indeed, the activity of both ERK and p38 MAPK demonstrated as p-ERK/ERK and p-p38/p38, respectively, in B cells were obviously enhanced, while JNK (p-JNK/JNK) was inhibited by the treatment with butyrate for 48 h as well as HDACi vorinostat (Fig. 7E, F). ERK and p38 MAPK was also enhanced, but JNK was not inhibited by the treatment of NaBu for only 1.5 h (Figure S6A). It, although preliminary, suggests that inhibition of JNK by NaBu for 48 h may be the result of upregulation of p38 MAPK since p38 inhibitor treatment for 48 h significantly increased the JNK activity (Fig. 7E, F) and inhibition of JNK by NaBu is slower than the activation of p38 MAPK. The hypothesis is strengthened by the result that treatment of JNKi alone for 48 h could enhance the B10% when cells were cultured with LPS (Figure S6B). Moreover, inhibition of the ERK and p38 MAPK, but not JNK, reduced B10 cell upregulation induced by butyrate (Fig. 7G). The above results suggest that butyrate as well as the other HDACis promotes B10 cell generation through the activation of p38 MAPK signaling pathway.

## Discussion

### The linkages among gut microbiota, SCFAs, Bregs, and diseases

Gut microbiota connects external signal and immune system closely, in which SCFAs act as essential mediators and modulate immune responses or metabolic homeostasis of the host^29^. In autoimmune diseases, SCFAs could induce Treg differentiation or suppress Th1/17 cell expansion^14,30^. For the effects of SCFAs on B cells, most investigations have been focused primarily on antibody production. Previous literature has reported that gut microbiota metabolite acetate, but not butyrate, can govern the differentiation of B cells into immunoglobulin A-producing plasma cells against pathogen infection^31^. A recent study has demonstrated that gut microbiota is essential for Breg differentiation^16^. *Clostridium butyricum*-derived butyrate induced IL-10 expression and switched the plasma cell differentiation to Breg development possibly via inhibiting HDAC1 activation and promoting Bcl6 expression, thus enforcing the suppressive effect of specific immunotherapy on intestinal allergic inflammation^18^. *Clostridium butyricum* in combination with specific immunotherapy was further proved to convert antigen-specific B cells to Bregs in asthmatic patients^32^. A recent study reported that pentanoate enhanced IL-10 secretion in Bregs in a manner dependent on glycolysis and p38 MAPK activity, and pentanoate-treated Bregs protect mice from experimental colitis and multiple sclerosis^19^. It is also reported that butyrate promotes B10 cell differentiation in non-obese diabetic mice and alleviates Sjogren’s syndrome through retinoic acid-related orphan receptor alpha^17^. In another recent study, SCFA butyrate could promote B10 cell function and control B cell differentiation partly via an AhR-dependent transcriptional program, but butyrate supplementation in mice does not alter the frequency or number of splenic Breg subsets^20^. It is not yet clear whether the supplementation of SCFAs could alter the frequency of Bregs in other tissues or peripheral blood in mice without immune deficiency. To clarify the effects of SCFAs on B10 cell generation and function, we induced B10 cells with different stimuli, which stimulate BCR, TLR9, or TLR4 signaling pathways in the presence or absence of SCFAs in vitro, and examined the induction of B10 cells by SCFAs in either healthy mice or in mice with acute or chronic inflammation. The results show that SCFAs could promote murine and human B10 cell generation in vitro in all stimulating conditions, and could enhance the B10 cell frequency in various tissues of different conditioned mice.

Murine B cells primed with LPS are capable of differentiating into B10 cells more effectively than CD40 mAb, especially under the existence of butyrate. In contrast, human B cells contain lower IL-10^+^ B cells when primed with LPS as they express little TLR4 receptor^33^. A previous study has reported that SCFA pentanoate induced B10 cells more effectively and caused less cytotoxicity than butyrate when stimulated by CpG-oligodeoxynucleotide (ODN) 2395^19^. The differences in B10 cell induction are related to the dosage of SCFAs (5 vs 0.5 mM) and different stimuli (CpG vs LPS). When the dosage decreased to a suitable level, butyrate did not cause cell death significantly, but enforced B10 generation more effectively compared to other SCFAs. The higher B10 cell induction efficiency by SCFAs, when stimulated with LPS, suggests that the immune system and gut microbiota evolve to respond efficiently to suppress the inflammation caused by pathogens like bacteria. Moreover, the effect of inducing B10 cells by SCFAs may be a result of the evolving interaction between gut microbiota and immune cells. The SCFAs produced by bacteria could suppress the host immune responses by induction of B10 cells, thus making the bacteria survive better.

### Application potential of modulating B10 cells by drugs like SCFAs and HDACi in the therapy of inflammatory and autoimmune diseases

Abnormalities of Bregs have been reported in inflammatory and autoimmune diseases. IL-10-producing Bregs decreased in mice with colitis^34^. The frequency of B10 cells was reduced in rheumatoid arthritis (RA) patients and inversely correlated with RA severity^35^. B10 cells can promote the differentiation of naive CD4^+^ T cells into Foxp3^+^ T cells, which are mainly in an IL-10-dependent manner or via cell contact mechanisms^36,37^. B10 precursor cells from RA patients showed impaired induction efficiency of Tregs than those from healthy controls^35^. A recent study has reported that patients with RA have lower stool butyrate, which is correlated with reduced IL-10^+^ Breg percentage^20^. Endogenous IL-10-producing Bregs were shown to play an important regulatory role in the induction and progression of CIA in mice^10^. Furthermore, the clinical symptoms are improved after treatment with butyrate-producing bacteria in combination with specific immunotherapy by educating B10 cells in asthmatic patients or allergic mice^18,32^. These evidences suggest that modulating Breg cells by SCFAs is a promising strategy for the treatment of diseases^4^. Growing evidence suggests that supplementation of dietary fiber or SCFAs is beneficial for the mitigation or prevention of autoimmunity disorders, such as experimental autoimmune encephalomyelitis, type 1 diabetes, and inflammatory bowel disease in mice^14,15,38,39^, of which SCFAs were often supplemented in drinking water at 150–200 mM or through intraperitoneal injection in 1 g/kg^19,30,40^. Indeed, it was recently reported that supplementation with butyrate increases serotonin-derived 5-hydroxyindoleacetic acid, activates AhR^+^ Bregs, and suppresses arthritis severity in a mouse model^20^, although the frequency of splenic Breg subsets is not altered by butyrate. Consistently, our study demonstrates that supplementation of butyrate suppressed both chronic inflammations modeled as CIA and acute inflammation modeled as DSS-induced colitis. It is interesting to note that, in our study, supplementation of NaBu or NaPe enhanced B10 cell frequency in B cells from the peripheral blood of healthy mice and peritoneal cavity fluids of mice with DSS-induced colitis, but not splenic B cells in CIA mice or mice with DSS-induced colitis. However, after culturing with CD40 mAb for 48 h for B10 cell maturation, splenic B cells from CIA mice treated with NaBu or NaPe showed higher B10 cell frequency than those from CIA mice without SCFA treatment, suggesting that supplementation of SCFAs primed B10pros.

Clinical application with Breg treatment in autoimmunity or inflammatory disorders has not been approved. The very low frequency of Bregs in the peripheral blood is one of the major obstacles in the clinical application of Breg. It requires ex vivo expansion of a large number of autologous Bregs without losing their regulatory function during the expansion. It has been reported that B10 cells induced by CD40 mAb^10^ or CpG^19^ inhibited arthritis in animal models, suggesting that in vitro induced Bregs could be applied for the treatment of autoimmunity disease. In our study, we developed an efficient method for ex vivo expansion of B10 cells. B10 cell percentage is enhanced significantly from 2 to ~50% of B cells, which proliferate faster than the other treatments when stimulated with LPS and induced with butyrate (Fig. 2B). B10 cell number could be increased to ~30 times of the initial one in 2 days by this method (Figure S1D). Adoptive transfer of B cells pre-expanded in vitro with this method enhanced the higher frequency of Treg cells in mice and showed more suppression of colitis than those without butyrate pretreatment (Fig. 4D). Although it is unclear whether the therapeutic effect is mediated by IL-10 or not, our study may benefit the future clinical application of B10 cells in the immunotherapy of inflammatory and autoimmune diseases.

### Histone acetylation and MAPK pathway may be shared by Breg subsets for their development and differentiation

SCFAs modulate cellular responses via the activation of GPCR or inhibition of HDAC. GPR41 and GPR43 are the most common receptors of SCFAs and their mRNA expression levels in B lymphocytes were lower than neutrophils or epithelial cells^41^. Acetate has a higher affinity to GPR43, but lower B10 cell induction efficiency than butyrate^42^. Furthermore, B10 cell generation induced by butyrate could not be mimicked by GPR43 agonist. Taken together, we exclude the possibility that GPR43 participants in SCFA promoted Breg differentiation. Conversely, the efficacy of Breg induction by SCFAs is consistent with cellular HDAC inhibitory potential regardless of stimulus condition^27^, which indicated that SCFAs might induce B10 cell generation through its inherent HDAC inhibitory activity. Indeed, HDACis mimicked the induction efficiency of SCFA, and histone acetytransferase (HAT) inhibitor reversed it partially, suggesting that histone acetylation is important for B10 cell generation. Our study also brings HDACis under the spotlight with regards to its application in the modulation of B10 cells in vitro or in vivo in the therapy of autoimmune and inflammatory diseases.

Further study with RNA-seq analysis identified the DEGs. PI3K-Akt and MAPK signaling pathways were enriched by the KEGG pathway in butyrate treatment, of which the PI3K-Akt pathway has been reported previously to be important for IL-10 production in Bregs when stimulated with CpG^21,43^. ERK and p38 pathway activation was reported to guide IL-10 production in macrophages and in B cells responding to LPS or CpG stimulation^9,44,45^. Consistently, our results show that blocking ERK or p38 MAPK pathway impaired butyrate-promoted B10 cell generation, and NaBu enhanced the phosphorylation of ERK1/2 and p38, but reduced the phosphorylation of JNK in B10 cells. Interestingly, p38 inhibitor significantly enhanced the JNK activity and JNK inhibitor enhanced B10%. Our study, although preliminary, indicates that activation of p38 MAPK and inhibition of JNK might be responsible for B10 cell induction by butyrate.

It is a long mysterious question how Breg cells develop, differentiate, and mature. The main obstacle to get the answer is the lack of unique cell surface markers or transcription factors exclusively identifying Breg^46^, although 90% of murine B10 cells could be distinguished by CD9. Our study sheds light on the mechanism of B10 cell development. SCFAs, especially butyrate, unfolded the structure of the chromosome near the transcription site or promoter site of IL-10 and other factors mediated by its inherent HDAC inhibitory activity, and increased the phosphorylation of ERK and p38 MAPK while inhibiting the phosphorylation of JNK. As a result, the changes in chromosome structure may contribute to the differentiation and function of Bregs, which needs to be further validated.

## Materials and methods

### Animal

Six- to 12-week-old healthy mice (C57BL/6J background, specific pathogen-free (SPF)) were purchased from the Experimental Animal Center of Sun Yat-sen University; DBA/1J mice (4–5 weeks old) were purchased from Beijing Vital River Laboratory Animal Technology Co., Ltd (Beijing, China). Mice were kept in individually ventilated cages in an SPF environment with a 12 h light–dark cycle at a constant temperature of 25 °C. The mice were acclimated for 7 days in this new environment prior to the formal experiment. The cages instead of animals are randomly divided into different groups because mice from different cages may fight with each other. The severity of the diseases was recorded by investigators who were blinded to the group allocations. Investigators were not blinded to the ex vivo experiments of cells from the animals. The experimental procedures employed in this study were approved by the Ethical Committee of Sun Yat-sen University (Approved Nos. 2019-3138, 2019-683, and 2020-0698).

### B10 cell induction by SCFAs in healthy mice treated with antibiotics

Healthy C57BL/6J mice were treated for 1 week with vancomycin (500 mg/l, MCE), neomycin (1 g/l; MCE), and metronidazole (1 g/l; MCE) in their water, and then fed with or without 150 mM acetate, propionate, butyrate, or pentanoate in drinking water containing antibiotics for additional 21 days before being sacrificed for sampling. SCFAs were usually supplemented in drinking water at 150–200 mM^30,38,40^ or through daily intraperitoneal injections in 1 g/kg^19,47^. To reduce the unnecessary animal usage, we adopted the dosage of SCFAs at 150 mM in the drinking water.

### Induction and treatment of colitis

To induce colitis, C57BL/6J mice were treated with DSS solution (2.5%; molecular weight 35–45,000 kDa; MP Biomedicals) in a drinking water bottle for 7 days and was replaced with regular drinking water for additional 3 days. For colitis treatment with NaBu, mice received an intraperitoneal injection with phosphate-buffered saline (PBS) or 1000 mg NaBu/kg mice daily throughout the duration of the experiment. For colitis treatment with adoptive transfer of B cells induced by NaBu, 2 × 10^6^ of B cells washed with PBS twice after cultured with LPS in the presence or absence of NaBu for 48 h were injected intraperitoneally into the recipient mice on days 1, 3, and 5 after colitis induction. Mice were sacrificed on day 10 and tissues were collected. Body weight and clinical disease severity were monitored daily, and the disease severity was scored according to a published scoring system^48^.

Colon tissue was flushed gently and segments were immediately fixed in neutral-buffered 10% formalin or frozen in liquid nitrogen and stored until use at −80 °C. After paraffin embedding, the 5-μm-thick sections were stained with hematoxylin and eosin and examined by Aperio AT2 scanner (Leica Microsystems). Histopathological evaluation was scored by two blinded observers using a previously published system:^48^ crypt architecture (0–3), degree of inflammatory cell infiltration (0–3), goblet cell depletion (0–3), and crypt abscess (0–1). The total histologic score was calculated as the sum of the individual variables and had a maximum score of 10.

### Induction and treatment of arthritis

The DBA/1J mice were randomly divided into five groups; three groups were given acetate, butyrate, or pentanoate in the drinking water containing 150 mM SCFA for 3 weeks before immunization and throughout the experiment thereafter. To induce arthritis with collagen, mice were immunized via intradermal injection with 100 μl emulsion made of 100 μg chicken type II collagen (Chondrex Inc., Redwood WA, USA) with an equal volume of complete Freund’s adjuvant containing 5 mg/ml heat-denatured *Mycobacterium tuberculosis* (Chondrex Inc.). A booster injection of 100 μg collagen emulsified in incomplete Freund’s adjuvant (Chondrex Inc.) was given in the same manner 3 weeks later. The arthritis severity of mice was evaluated every 3–4 days according to a previously published scoring system^49^. On day 32 post first immunization, splenocytes were prepared for the detection of B10 cell maturation after culturing with CD40 mAb for 48 h.

### B cell preparation, culture, and stimulation

Murine splenocyte suspensions were generated by gentle dissection and mashed through a 70 μm cell strainer, followed by erythrocytes depletion using red cell lysis buffer (Cowin Bio, Beijing, China). Total B cells were enriched by positive separation using anti-CD19 microbeads (Miltenyi Biotec)^46^ or negative selection using B Cell Isolation Kit (STEMCELL) according to the manufacturer’s instructions. Human PBMCs were prepared with Lymphocyte Isolation Kit (TBDscience, Tianjin, China) from the venous blood donated by the author Jianbo Sun. Cells were cultured with specific stimuli in a complete RPMI-1640 medium containing 10% fetal bovine serum (Hyclone), 1% penicillin/streptomycin (Corning), and supplemented with 1% nonessential amino acids (Corning), 1 mM sodium pyruvate (Corning), 1 mM l-glutamine (Corning), and 50 μM 2-mercaptoethanol (Thermo Fisher Scientific). Cells were plated at 0.5–3 × 10^6^ cells/ml and cultured at 37 °C with 5% CO_2_. For B10 cell analysis, splenocytes, PBMCs, and purified B cells were cultured with 1 μg/ml human or murine CD40 mAb (BioLegend), 40 nM CpG-ODN 1826 (Invitrogen), 4 μg/ml CpG-ODN 2395 (Invitrogen), or 10 μg/ml LPS (Sigma) for 2–4 days and stimulated with L + PIM for cytokine production at 5 h before cell harvest. In some experiments, cells were treated with inhibitors or metabolites, including HDACis TSA (1–10 nM; Beyotime) and vorinostat (100–500 nM; MCE), GPR41 agonist AR420626 (1–10 μM; APExBIO), GPR43 agonist (1–10 μM; Sigma-Aldrich) or antagonist GLPG0974 (500 nM; Sigma-Aldrich), GPR109A agonist niacin (1 mM; Sigma-Aldrich), HAT inhibitor anacardic acid (1–10 μM; MCE), ERK1/2 inhibitor U0126 (5 μM; MCE), p38 inhibitor SB 203580 (5 μM; MCE), or JNK inhibitor SP600125 (1 μM; MCE).

### Cell proliferation assay

A total of 1 × 10^7^/ml B cell-depleted splenocytes or purified B cells resuspended in PBS were labeled with 2 μM CFSE (BioLegend) for 15 min at 37 °C and then washed twice with complete RPMI-1640 medium for culture. To monitor the polarization of B cells, murine splenic B cells were isolated and labeled with CFSE and then cultured with CD40 mAb, LPS, or CpG-ODN 1826 in the presence or absence of 0.5 mM NaBu for 48–96 h plus L + PIM for the last 5 h. To monitor T cell proliferation, splenocytes labeled with CFSE were seeded in plates precoated with αCD3 (5 μg/ml; BioLegend) and αCD28 (1 μg/ml; BioLegend) and cocultured for 72 h with B cells (splenocytes: B cells = 1:1 or 1:2), which were washed twice with PBS after induced by LPS with or without NaBu for 48 h. Cell proliferation was detected by dilution of CFSE with flow cytometry.

### Cell staining for flow cytometry

Flow cytometry is used to detect the proteins labeled with the following antibodies conjugated with specific fluorochromes from BioLegend: CD16/32 (93), CD19 FITC or AF700 (6D5), CD4 PE/Dazzle (GK1.5), CD8a BV605 (53-6.7), CD9 PECy7 or AF647 (MZ3), CD11b APC (M1/70), Ly6G PECy7 (RB6-8C5), IL-10 FITC or PE (JES5-16E3), IL-6 APC (MP5-20F3), TNF-α PE (MP6-XT22), IFN-γ PerCP/Cy5.5 (XMG1.2), and FOXP3 AF647 (150D). LIVE/DEAD Fixable Near-IR Dead Cell Stain Kit was purchased from Invitrogen. Briefly, for extracellular multicolor analysis, cells were incubated with CD16/32 Fc block and live/dead discrimination dye on wet ice in the dark for 20 min. After that, cells were washed with PBS and stained with surface markers for 30 min in the dark. For intracellular cytokine analysis, cells were stimulated with L + PIM during the last 4–6 h culture and then harvested for staining with surface markers, followed by intracellular fixation and permeabilization with Cytofix/Cytoperm Fixation/Permeabilization Solution Kit (BD) and incubation with intracellular Abs. The staining procedure of intranuclear Foxp3 expression is the same as intracellular cytokine staining, except that the cells were fixed and permeabilized with True-Nuclear™ Transcription Factor Buffer Set from BioLegend other than from BD. Flow cytometric data were acquired on Fortessa (BD) or Cytoflex (Beckman). Data were analyzed using the FlowJo 10.0.7 software (TreeStar).

### Calcium influx assay

Bone marrow was isolated from mice femur and tibia and lysed to deplete red blood cells. A single-cell suspension was stained with CD11b APC and Ly6G-PC7 following Fc block and live/dead discrimination on ice. After that, the cell suspension was loaded with Fluo-4 AM (2 μM) in Hank’s balanced salt solution buffer with calcium and magnesium for 45 min at room temperature, then washed twice, and finally incubated for 20 min. Samples were treated with GLPG0974 (10 μM) or equilibrated at room temperature for 15 min before the analysis. Ca^2+^ influx data were acquired for the indicated neutrophil subsets by BD LSRFortessa flow cytometry and analyzed with FlowJo. The stimulus was added with a pipette after baseline was recorded for 60–100 s.

### HDAC activity assay

CD19^+^ B cells were isolated from the spleen of healthy mice and polarized to B10 cell differentiation by culture with 10 μg/ml LPS and at an indicated concentration of SCFAs. The nuclear protein was then extracted and the HDAC activity was measured using BioTek according to the manufacturer’s instructions (EpiGentek). Briefly, the nuclear protein was incubated with biotinylated HDAC substrate coated onto the wells for 1 h; the remaining undeacetylated substrate was captured with a high-affinity antibody and then detected with detection antibody in ELISA.

### Quantitative reverse transcription-PCR

Total RNA was extracted from B cells or colon tissue using TRNzol reagent (TIANGEN, Beijing, China) and RNA quantification was performed on Nanodrop 2000. RNA (0.5–1 μg) was reverse transcribed using the cDNA Synthesis Kit (Yeasen Biotech, Shanghai, China) according to the manufacturer’s instructions. The qPCR reaction was performed using the SYBR Green Kit (Yeasen Biotech) and on QuantStudio 5/7 (Applied Biosystems). Primers were designed and synthesized with the sequences listed in Table 1.

**Table 1:** Primer list for RT-qPCR.

Prime names	Primer (5′–3′)	Sequence
*Actb*	Forward	ACCAGAGGCATACAGGGACA
	Reverse	CTAAGGCCAACCGTGAAAAG
*Gapdh*	Forward	AGGTCGGTGTGAACGGATTTG
	Reverse	GGGGTCGTTGATGGCAACA
*Il10*	Forward	TTTGAATTCCCTGGGTGAGAA
	Reverse	GGAGAAATCGATGACAGCGC
*Prdm1*	Forward	AAGACGTTCGGTCAGCTCTCCA
	Reverse	CTGGCACTCATGTGGCTTCTCT
*Ffar3*	Forward	CTTCTTTCTTGGCAATTACTGGC
	Reverse	CCGAAATGGTCAGGTTTAGCAA
*Ffar2*	Forward	CTTGATCCTCACGGCCTACAT
	Reverse	CCAGGGTCAGATTAAGCAGGAG
*Gpr109a*	Forward	CTGTTTCCACCTCAAGTCCTGG
	Reverse	CATAGTTGTCCGTCAGGAACGG
*Il6*	Forward	CACTTCACAAGTCGGAGGCT
	Reverse	CTGCAAGTGCATCATCGTTGT
*Il1b*	Forward	TGCCACCTTTTGACAGTGATG
	Reverse	TGATGTGCTGCTGCGAGATT
*Ifng*	Forward	CAGCAACAGCAAGGCGAAAAAGG
	Reverse	TTTCCGCTTCCTGAGGCTGGAT
*Tnf*	Forward	GGTGCCTATGTCTCAGCCTCTT
	Reverse	GCCATAGAACTGATGAGAGGGAG

### RNA-seq and data analysis

Naive B cells were induced for Breg differentiation with 10 μg/ml LPS in the absence or presence of 0.5 mM NaBu for 48 h. Total RNA was purified as described above. mRNA was enriched with oligo(dT) attached to magnetic beads and fragmented into small pieces, cDNA library was generated using random hexamer-primed reverse transcription, followed by second-strand cDNA synthesis, and sequenced on an MGI 2000 platform (BGI, Shenzhen, China). The clean reads were mapped to the *Mus musculus* reference genome (mm10) with STAR v1.5.1. Gene expression levels were quantified using HTSeq-count v0.11.3, followed by the analysis of DEGs using the default settings of the DEseq2 v1.28.1 algorithm; significant DEGs were set at a false discovery rate of <0.05 and a fold change of >2×. Subsequent processing and visualization of the data were completed in RStudio or TBtools^50^. The GSEA was conducted with GSEA v4.0.2 (Broad Institute)^51^. The RNA-seq data are deposited in the GEO database (access no. GSE166021).

### Western blotting

Antibodies included GAPDH (AB0037, Abways), Erk1/2 (137F5, CST), pErkThr202/Tyr204 (D13.14.4E, CST), p38 (D13E1, CST), p-p38Thr180/Tyr182 (D3F9, CST), JNK (9255, CST), and p-JNK-Thr183/Tyr185 (81E11, CST). For western blotting, purified B cells were harvested, resuspended in RIPA buffer containing 1× protease/phosphatase inhibitor cocktail, and rotated at 4 °C for 30 min. Lysates were centrifuged at 12,000 r.p.m. for 15 min at 4 °C, and the whole protein extraction was collected from the supernatant. In total, 10–30 μg protein per sample was resolved by sodium dodecyl sulfate-polyacrylamide gel electrophoresis, transferred to polyvinylidene fluoride membranes, and blotted using the designed antibody. Bound antibodies were developed with horseradish peroxidase-conjugated secondary antibodies using the ECL substrate (Merck).

### Statistical analysis

All data were analyzed with GraphPad Prism 9. Differences between two or more groups were analyzed, respectively, by the Student’s *t* test or analysis of variance multiple comparisons. Data shown are presented as means ± SD.

## Supplementary information

Supplementary materials

supplemental table

## Data Availability

All data generated or analyzed during this study are included in this published article and its Supplementary information files.
