# Supplementary material for: Effects of short-chain fatty acids in inhibiting HDAC and activating p38 MAPK are critical for promoting B10 cell generation and function
Source: Cell Death Dis. 2021 Jun 7;12(6):582. doi: 10.1038/s41419-021-03880-9 (PMC8184914; doi:10.1038/s41419-021-03880-9)
Supplement: Supplementary file 1 — Supplementary materials [file 41419_2021_3880_MOESM1_ESM.pdf]

Supplementary Information

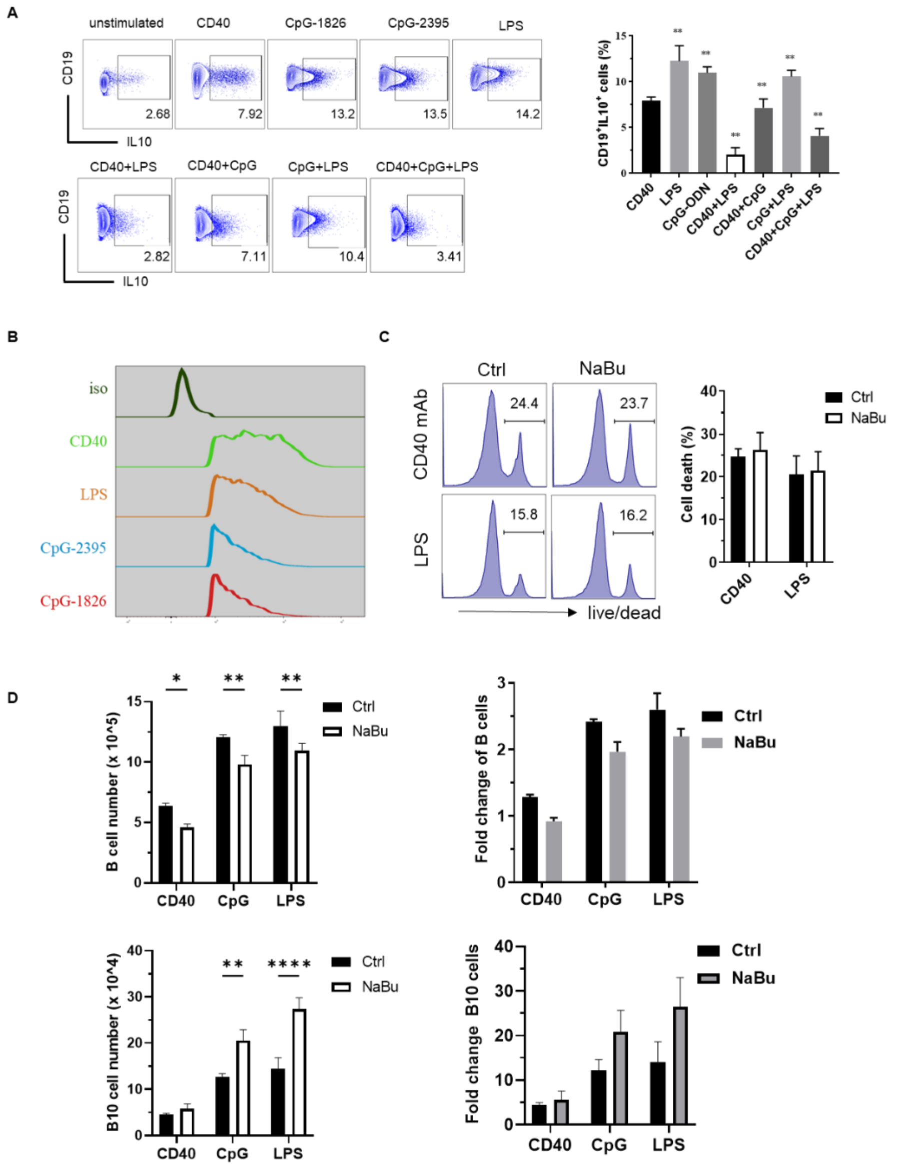

Fig. S1 The effects of SCFAs and different stimuli on the frequency, proliferation and death of B10 cells. (A) Representative FACS plots of splenic B cells purified from C57BL/6 mice and cultured with murine CD40 mAb, LPS, CpG-1826, CpG-2395, and the combined stimuli for 48h. The bar graph is a statistical result of B10 cell percentage and presented as mean ± SD from three independent experiments. **p* < 0.05, ***p* < 0.01 compared to CD40 mAb. **(B)** The mean fluorescence intensity of IL-10 in $CD19^+$ cells. **(C)** Representative FACS plot of cell death and the statistical results. **(D)** The increase of B cell and B10 cell number under the treatments of CD40, CpG-ODN or LPS in the presence or absence of NaBu for 48h. The initial number of purified B cells was 5 x 10^5. Total B cells were counted and B10 cells were calculated by the frequency of total B cells. The initial number of B10 cells is assumed as 2% of the initial total B cells for calculation of the fold change of B10 cells after 48h stimulation. **p* < 0.05, ***p* < 0.01 compared between Ctrl and NaBu.

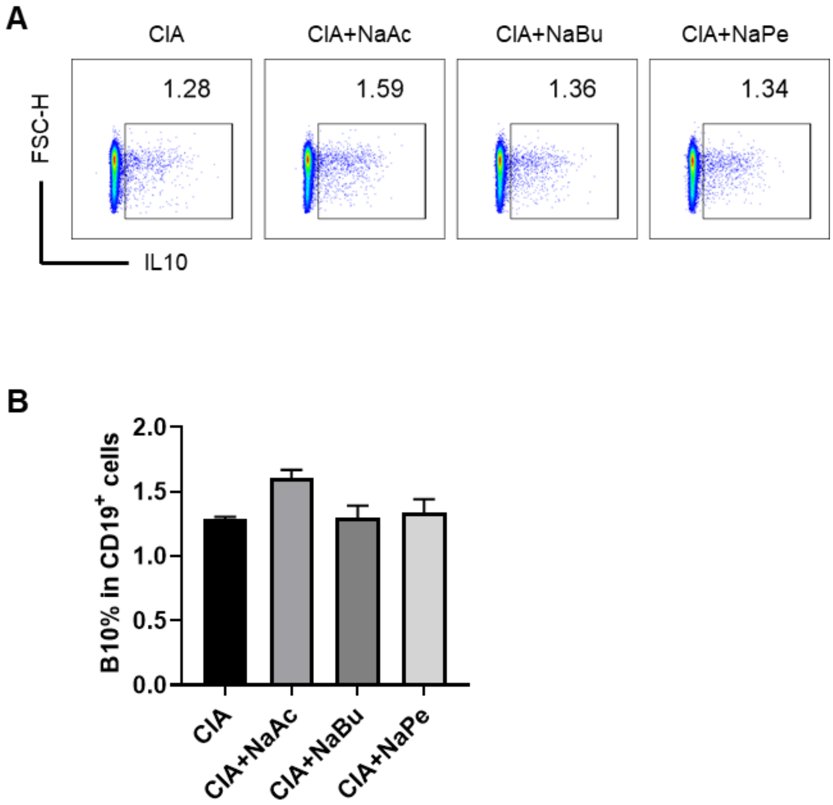

Fig. S2 Splenic B10 cell frequency in CIA mice. (A) Representative FACS plots of IL-10^{+} cells in CD19^{+} B cells from splenocytes of mice treated as same as Figure 3C. Cells were cultured with L+PIM for 5h before staining. **(B)** Bar graph of the statistical result of B10 cell percentage in (A). The data are presented as mean ± SD from three independent experiments.

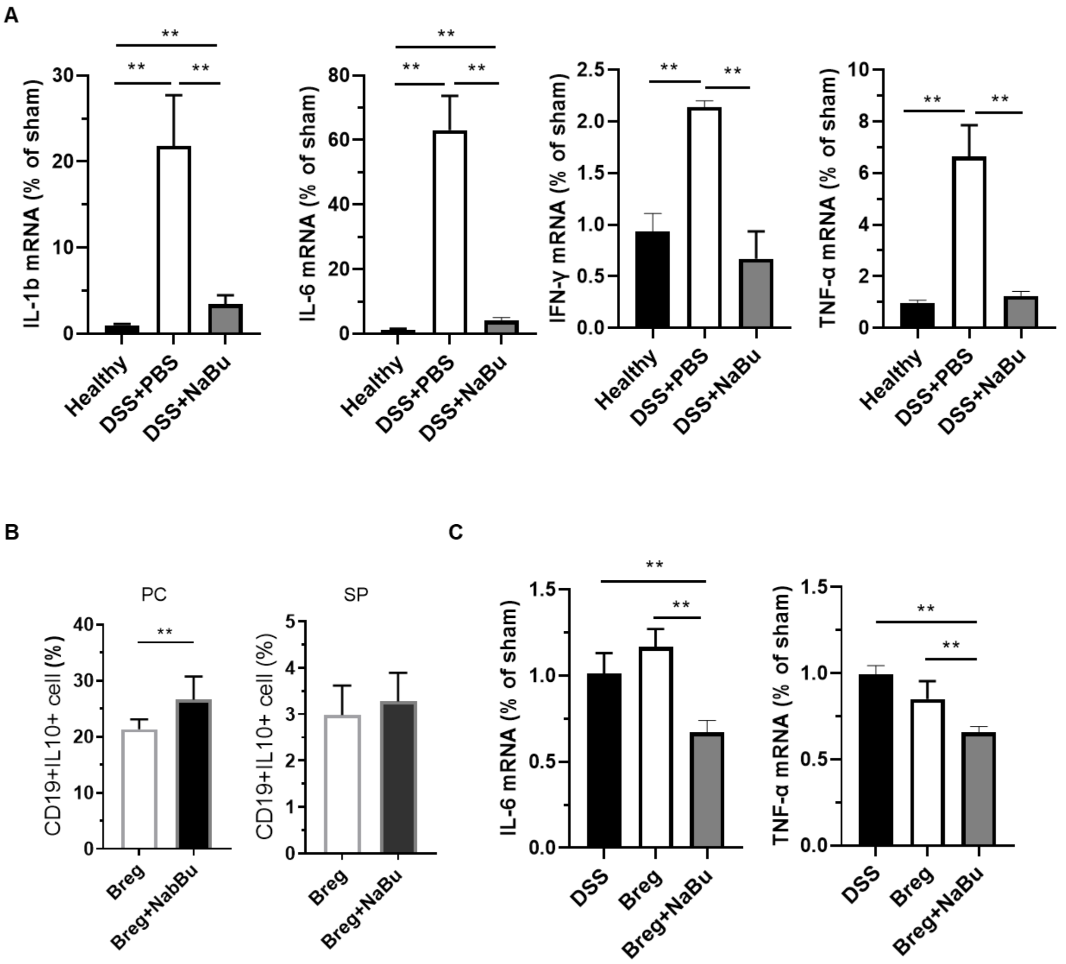

Fig. S3 Butyrate intervention maintain the potential of B10 cells and down-regulated the mRNA expression of inflammatory cytokines. (A) The relative mRNA expression of the inflammatory cytokines in colon tissue homogenates of healthy or DSS-induced mice with or without treatment of sodium butyrate (n=4). **(B)** The frequency of B10 cells from the host mice on day 10 same as those in Figure 4D. **(C)** The relative mRNA expression of IL-6 and TNF-α in colon tissue homogenates of DSS-induced mice with or without adoptive transfer of Breg cells that were pretreated with or without NaBu (n=4). $**p < 0.01$.

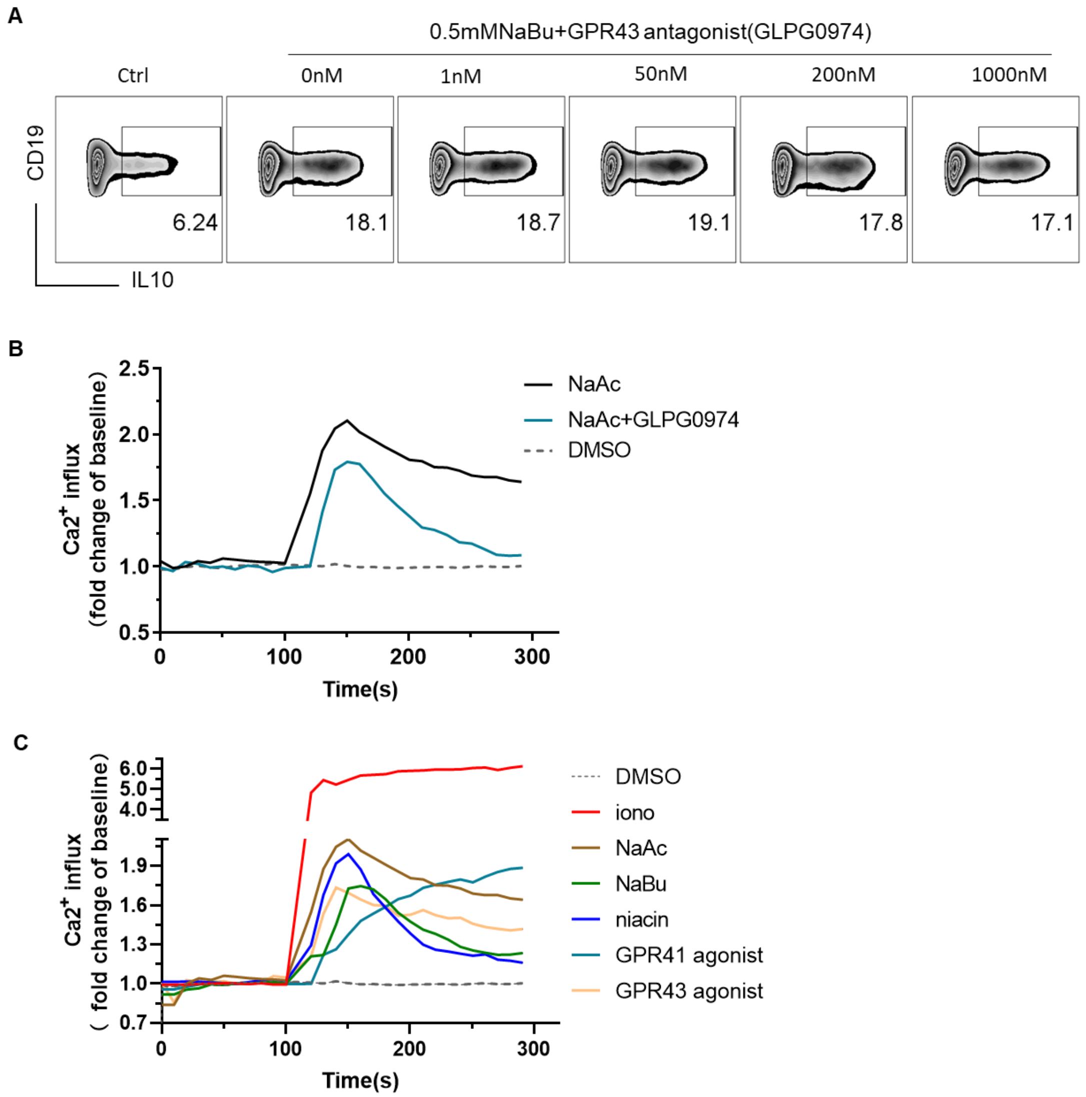

Fig. S4 Activities of GPCR agonist or antagonist. (A) The inhibitory activity of GPR43 antagonist (GLPG0974) on B10 cell generation evaluated by titration. **(B)** The calcium influx in bone marrow cells induced by NaAc was inhibited by GLPG0974. **(C)** The activation of GPCR agonist were evaluated by calcium influx assay with bone marrow cells. The stimuli in (B-C) include sodium acetate (1mM), sodium butyrate (1mM), niacin (1mM), GPR41agonist (10μM), and GPR43 agonist (10μM).

A

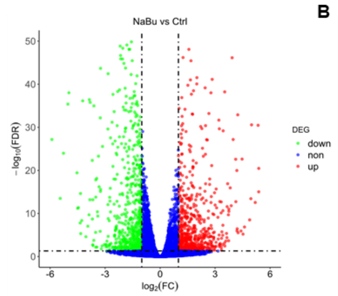

B

Gene Symbol	log2FoldChange	pvalue	padj
Il10	2.478229	2.48E-89	2.11E-86
Cd9	1.846531	1.71E-14	7.09E-13
Ffar2	2.38206	1.27E-06	1.93E-05
Ahr	2.006496	1.28E-44	2.95E-42
Prdm1	-2.36736	5.42E-11	1.52E-09
Il6	-4.45878	8.81E-90	7.74E-87
Tnf	0.334028	0.454579	0.720105

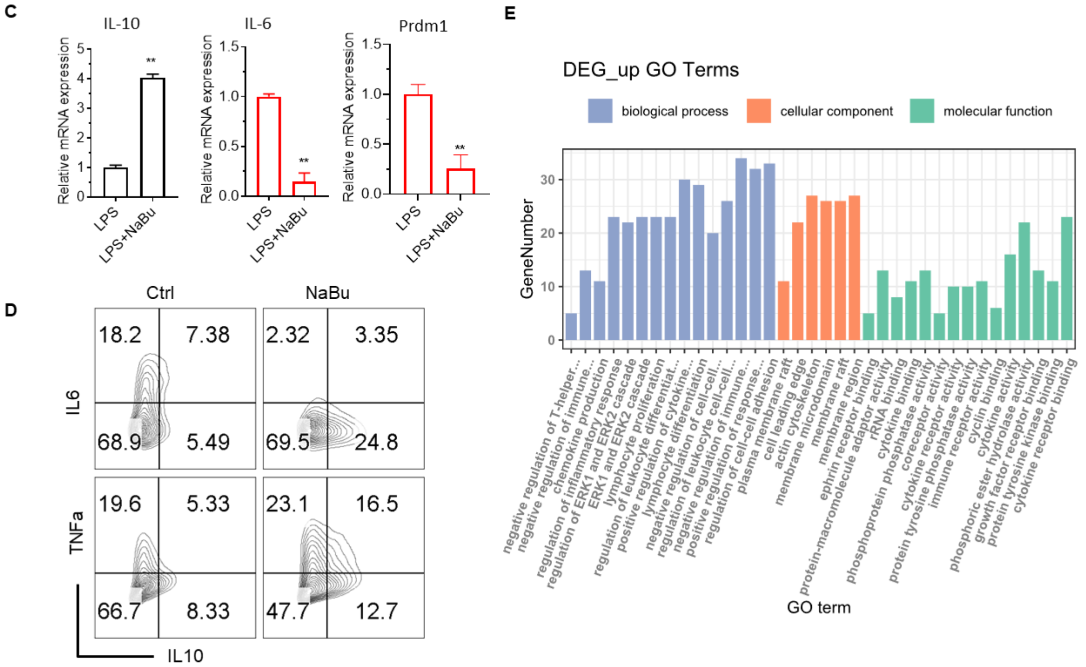

Fig. S5 Differentially expressed genes identified by RNA-seq analysis and their validation by RT-qPCR and flowcytometry. (A) Differentially expressed genes (fold change>2 and p-value of < 0.05) were visualized as volcano plot. **(B)** Fold change of expression of several genes derived from RNA-seq data. **(C-D)** Relative mRNA level of IL-10, IL-6 and Prdm1 detected by RT-qPCR (C) and protein level of IL-6 and TNFα measured by flowcytometry (D) in B cells treated as same as the RNA-seq samples for validation of RNA-seq data. **(E)** GO enrichment analysis of up-regulated DEGs. The data are presented as mean ± SD from three independent experiments. $^{**}p < 0.01$ compared to LPS.

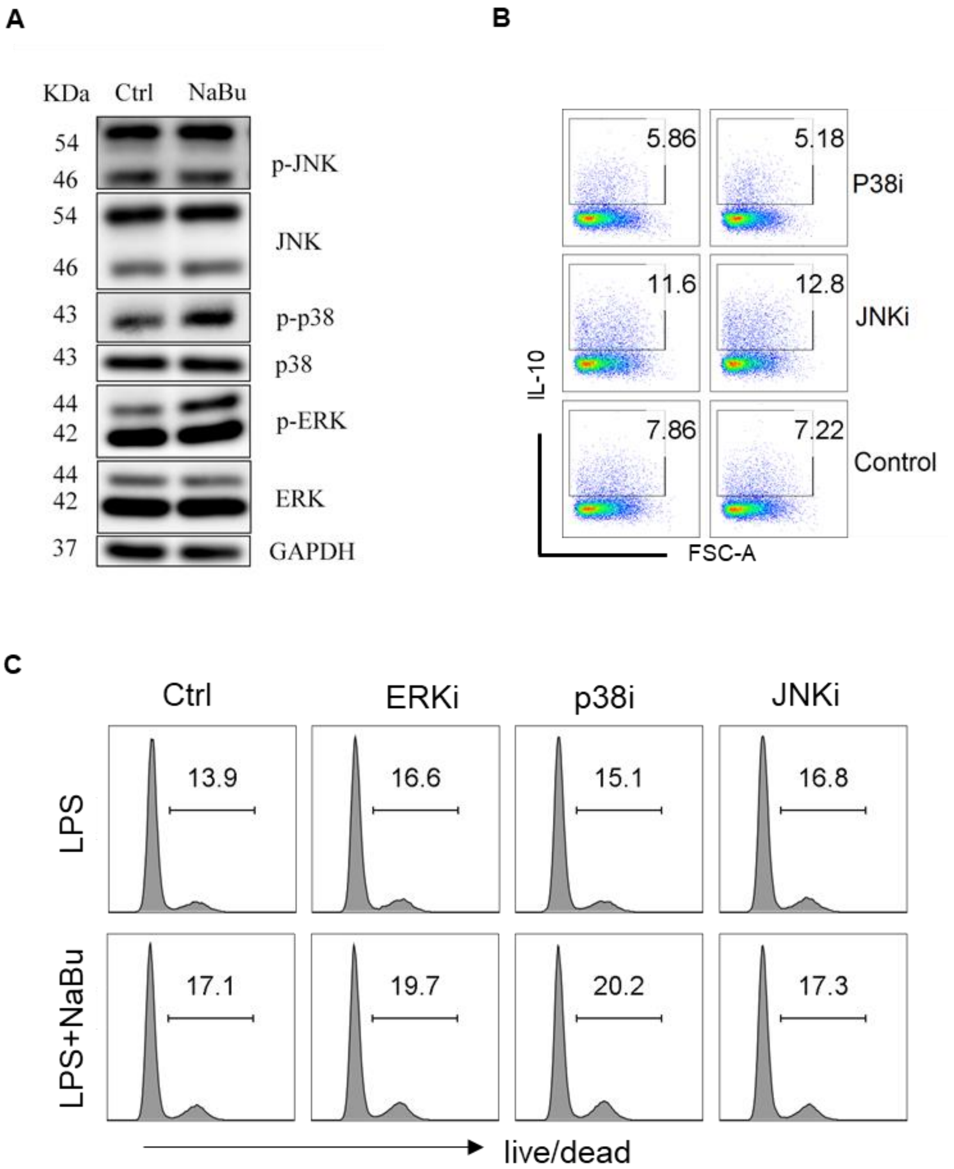

Fig. S6 Roles of NaBu on MAPK pathway and of inhibitors on B10 cell induction and cell death. (A) Protein level visualized by immunoblotting in B cells cultured for only 1.5h in the presence of LPS with or without NaBu (0.5mM). **(B)** Representative B10 cell frequency in B cells cultured for 48h in the presence of LPS with or without inhibitors. **(C)** Representative FACS plot of the percentage of dead cells. The concentration of inhibitors: ERKi (5 μM), p38i (5 μM) and JNIKi (1 μM).

Table S1. Full list of differentially expressed genes.
